# PRyMordial: the first three minutes, within and beyond the standard model

**DOI:** 10.1140/epjc/s10052-024-12442-0

**Published:** 2024-01-26

**Authors:** Anne-Katherine Burns, Tim M. P. Tait, Mauro Valli

**Affiliations:** 1 Department of Physics and Astronomy, University of California, Irvine, CA 92697 USA; 2 C.N. Yang Institute for Theoretical Physics, Stony Brook University, Stony Brook, NY 11794 USA; 3 INFN Sezione di Roma, Piazzale Aldo Moro 2, 00185 Rome, Italy

## Abstract

In this work we present PRyMordial: A package dedicated to efficient computations of observables in the Early Universe with the focus on the cosmological era of Big Bang Nucleosynthesis (BBN). The code offers fast and precise evaluation of BBN light-element abundances together with the effective number of relativistic degrees of freedom, including non-instantaneous decoupling effects. PRyMordial is suitable for state-of-the-art analyses in the Standard Model as well as for general investigations into New Physics active during BBN. After reviewing the physics implemented in PRyMordial, we provide a short guide on how to use the code for applications in the Standard Model and beyond. The package is written in Python, but more advanced users can optionally take advantage of the open-source community for Julia. PRyMordial is publicly available on GitHub.

## Introduction

The snapshot of the Universe approximately three minutes after the Big Bang [1] can be regarded as one of the most remarkable predictions of the Standard Model (SM) of Particle Physics in conjunction with the (so-called) concordance model of Cosmology, $\Lambda$CDM.

While a theory for the origin of chemical elements based on an epoch of high-energy densities and pressures was already formulated by Alpher, Bethe, and Gamow more than seventy years ago [2], the discovery of the quasi-black body spectrum of the Cosmic Microwave Background (CMB) [3, 4] paved the road for the modern formulation of the theory of Big Bang Nucleosynthesis (BBN) [5]. Indeed, thanks to the CMB, we know today that the SM particle species were in a thermal state during an epoch dominated by radiation. Extrapolating this cosmological picture back in time when the Universe was not yet transparent to light, within the standard lore of Cosmology and of Particle Physics we can accurately predict [6–11]:

1. The evolution of the number of relativistic degrees of freedom until recombination, $N_{\textrm{eff}}$;
2. The cosmological abundance of light nuclides synthesized from protons and neutrons, as a function of the number density of baryons relative to photons, $\eta _{B} \equiv n_{B}/n_{\gamma }$.

Regarding *1)*, given the current knowledge of neutrino oscillations [12], $N_{\textrm{eff}}$ is predicted in the SM via solving a set of integro-differential equations for the neutrino density matrix at finite temperature [13], yielding $N^\textrm{SM}_{\, \mathrm eff} = 3.044$ with an error estimated to be below the level of per mil [14–16].

Concerning *2)*, a detailed analysis of CMB anisotropies in temperature and polarization currently constrains $\eta _{B}$ with 1% accuracy or better [17], anchoring the primordial asymmetry between baryons and anti-baryons to be $\mathcal {O}(10^{-10})$ [18]. Assuming no large asymmetry in the lepton sector as well, see e.g. [19], standard BBN turns into an extremely predictive theory, often dubbed “parameter free”.

On the observational side, multi-wavelength astronomical campaigns have been able to provide rich spectroscopic information about emission and absorption lines of gas clouds in metal-poor extra-galactic environments, see e.g. [20–23], bringing us today to a percent-level determination of the abundance of primordial deuterium and helium-4. Given the predictions of the standard theory and the precision of those measurements, together with the strong constraints on the thermal history provided by the CMB [24, 25], the study of the Early Universe around the BBN epoch offers unique insight on New Physics (NP) [26–35].

Looking at the exciting prospects of next-gen CMB experiments [36–38], and at the expected future sensitivity in the field of observational astronomy [39, 40], it is therefore very timely to have tools at our disposal that allow for numerically efficient, yet precise computations that test the SM in the Early Universe, and that are flexible enough to broadly explore NP scenarios.

A few packages have already been developed to accurately investigate the BBN era. A publicly available version of the historical code of Ref. [41] (whose most up-to-date version is currently adopted by the PDG [42]) is described in [43]. At the same time, publicly released codes dedicated to state-of-the-art BBN analyses are also available; in particular:

- PArthENoPE [44–46] is a code originally written in FORTRAN 77 that in its latest re-incarnation also enjoys a graphical user interface; it offers a very efficient evaluation of BBN light-element abundances based on fitting formulae worked out for both weak rates and nuclear cross sections.
- PRIMAT [47, 48] is an user-friendly Mathematica package containing all the inputs and ingredients for an ab-initio computation of neutron freeze-out and of weak rates; moreover, it has tabulated the largest nuclear network at hand in order to track the abundance of heavy nuclides as well.

Both codes include a few built-in options to account for the study of some specific NP scenarios. AlterBBN [49, 50] is a C++ open-source software developed for broad investigation of Physics Beyond the SM (BSM) in the BBN era. However, while allowing for fast numerical evaluations, AlterBBN does not implement the level of detail and accuracy in its computation of light primordial abundances present in PArthENoPE or PRIMAT. In fact, these two packages may currently represent the best tools to perform precision cosmological analyses [24, 51].

While powerful and flexible, these public codes nevertheless suffer from a few limitations and/or missing features. A precision tool for Cosmology, able to handle BSM Particle Physics should:

- Allow for the evaluation of the physics of the thermal bath in a fast but precise way, following, e.g., the approach highlighted in  [34, 52, 53], and implemented in the standalone code  NUDEC_BSM;
- Interconnect a first-principle computation of the thermal background with an ab-initio precise calculation of the neutron-to-proton ($n \leftrightarrow p$) conversion, as the one implemented in PRIMAT  [47];
- Render easily accessible exploration of the impact of the input parameters characterizing the BBN era and the uncertainties in the set of thermonuclear rates on the basis of more model-dependent/more data-driven approaches available, see [35, 54, 55];
- Adopt a user-friendly, modern programming language compatible with numerical efficiency of the computations, while smoothly interfacing with standard libraries for statistically advanced analyses like Monte Carlo (MC) ones [56, 57], see e.g. [58–60].

In this work, we introduce PRyMordial: A new public tool for the community of Particle Physics and Cosmology that precisely aims at filling in the above gaps for precision studies on the physics of the Early Universe both within and beyond the SM. The package is written and runs entirely with Python 3. Moreover, for the most advanced users, the resolution of the set of stiff differential equations for the BBN nuclear-reaction network can be further optimized with the optional switch to some routines of the SciML kit [58], the open-source software for scientific machine learning in Julia.

Our article is organized as follows: In Sect. 2 we present all the key ingredients of the physics implemented in PRyMordial; In Sect. 3 we discuss in detail how PRyMordial is structured and we provide several examples on the usage of the code; In Sect. 4 we comment on future directions for further development of PRyMordial along with possible interesting applications. We finally collect in Appendix A a set of instructions for the installation of the package and its dependencies.

## Physics in PRyMordial

In this section we present the key equations present in PRyMordial, which stand out as a reference for the physics implemented within the code as well as representing a guideline regarding its use (see Sect. 3). We organize the presentation in three distinct topics: the thermodynamics of the plasma; the weak rates for $n \leftrightarrow p$ conversion; and the set of thermonuclear rates for the key reactions responsible of the non-zero primordial abundance of deuterium, helium-3 and -4, and lithium-7.

### Thermodynamics beyond instantaneous decoupling approximation

The description of the thermal background during the BBN era in $\Lambda$CDM follows from an isotropic, homogeneous Universe modelled by the Einstein field equation:

1 $$\begin{aligned} H^2 \equiv \left( \frac{d \log {a}}{dt} \right) ^2 = \frac{8 \pi }{3 M^2_{\textrm{Pl}}} \, \rho _\textrm{tot} \ , \end{aligned}$$

where *H* is the Hubble rate of space-time expansion, *a* the scale factor of the FLRW metric, $\rho _\textrm{tot}$ the total energy density present in the Universe, and $M_\textrm{Pl} \equiv 1/\sqrt{G_\textrm{N}}$, with $G_\textrm{N}$ the Newton gravitational constant.

Within an axiomatic characterization of the Early Universe provided by *local thermodynamic equilibrium* [59, 60], SM species are described according to the spin-statistics theorem and the temperature $T_{\gamma }$ of the thermal bath (provided chemical potentials $\mu$ can be neglected, i.e., $\mu /T_{\gamma } \ll 1$). Standard BBN takes place during radiation domination, and thus features contributions to $\rho _\textrm{tot}$ largely from relativistic species, i.e. $\rho _\textrm{tot} \simeq \rho _\textrm{rad} \propto T_{\gamma }^4$. This observation dramatically simplifies the investigation of BBN, allowing one to decouple the study of the thermal background from the nucleon dynamics. Indeed, after the QCD crossover takes place [61] nucleons are already non-relativistic, i.e. they are highly Boltzmann-suppressed well before the MeV scale temperatures characteristic of the BBN era.

Hence, for temperatures $T_{\gamma } < \mathcal {O}(10)$ MeV, one can accurately describe $\rho _{tot}$ in the SM as a sum of just three contributions:

2 $$\begin{aligned} \rho _{\gamma }= & {} \frac{\pi ^{2}}{15} \, T_{\gamma }^4 \, \ \rho _{\nu , \mathrm tot} = 3 \, \rho _{\nu } = \frac{7 \pi ^{2}}{40} \, T_{\nu }^4 \, \nonumber \\ \rho _{e^\pm }= & {} \frac{2}{\pi ^{2}} T^{4}_{\gamma } \, \int _{x_{e}}^{\infty } d \tilde{x} \, \frac{\tilde{x}^{2} \sqrt{\tilde{x}^2-x_e^2}}{\exp (\tilde{x}+1)} \, \end{aligned}$$

where $x_e \equiv m_e/T_{\gamma }$ and we distinguish the temperature of the electron-positron-photon system, $T_\gamma$, from that of neutrinos, $T_\nu$.^1^ Indeed, while the initial condition $T_\nu = T_\gamma$ must hold at early times for the two systems to be in thermal (more precisely, in chemical and kinetic) equilibrium, around the MeV scale neutrinos are expected to freeze out from the thermal bath as weakly-interacting relativistic species  [63]. Neglecting tiny departures from a Fermi-Dirac distribution in $\nu$ phase space, one can study the evolution of the two systems according to the momentum-integrated Boltzmann equations:

3 $$\begin{aligned} (\rho _{\gamma }^{\prime }+\rho _{e^{\pm }}^{\prime })\, \frac{dT_{\gamma }}{dt}= & {} - 4 H \, \rho _{\gamma } - 3 H (\rho _{e^{\pm }} + p_{e^{\pm }}) + \delta C_{e^{\pm }} \, \nonumber \\ \rho _{\nu , \mathrm tot}^{\prime } \, \frac{dT_{\nu }}{dt}= & {} - 4 \, H \, \rho _{\nu , \mathrm tot} + \delta C_{\nu } \, \end{aligned}$$

with $^\prime \equiv d/dT$, *p* the pressure density (equal to $\rho /3$ for a relativistic species), $\delta C$ the (momentum integrated) collision term, and where we have conveniently traded energy densities for temperatures in light of Eq. (2). Due to energy-momentum conservation, the sum over all $\delta C$s must vanish, so that one recovers the continuity equation for the total energy density of the Universe:

4 $$\begin{aligned} \frac{d \rho _\textrm{tot}}{dt} + 3 H (\rho _\textrm{tot} + p_\textrm{tot}) = 0 \ . \end{aligned}$$

In the SM, where Eq. (3) holds, such a constraint implies: $\delta C_{\nu } = -\delta C_{e^{\pm }}$. The collision term $\delta C_{\nu }$ has been evaluated in [63] under Maxwell-Boltzmann approximation, nicely refined in [52, 53] taking into account relativistic corrections as well as finite mass effects from $m_{e} \ne 0$, and more recently re-computed independently in [34]. Including finite temperature QED corrections to the electromagnetic plasma [64], one can solve the system of coupled differential equations in Eq. (3), to find $T_{\gamma }(t)$, $T_{\nu }(t)$, and, as a byproduct, $T_{\nu }(T_{\gamma })$.^2^ Such a treatment naturally includes non-instantaneous decoupling effects, and allows one to perform a numerically fast, but accurate prediction of the effective number of relativistic degrees of freedom from first principles, yielding (in the SM) at $T_{\gamma } \ll$ MeV:

5 $$\begin{aligned} N_\textrm{eff} \equiv \frac{8}{7} \left( \frac{11}{4} \right) ^{4/3}\left( \frac{\rho _\textrm{rad}-\rho _{\gamma }}{\rho _{\gamma }}\right) = 3.044 \ , \end{aligned}$$

while also opening up novel explorations of BSM physics in the Early Universe [33, 34, 53].^3^

Based on these results, one can also easily evaluate the relic density of neutrinos (neglecting phase space spectral distortions). From the CMB we know the photon temperature today is $T_{\gamma ,0} = 0.2348$ meV; plugging this value into the solution of Eq. (3) yields the temperature $T_{\nu ,0} = 0.1682$ meV, corresponding to the cosmological abundance of SM neutrinos:

6 $$\begin{aligned} \Omega ^\mathrm{(rel)}_{\nu } h^2= & {} \left( \frac{7 \pi ^{2}}{120} \, T_{\nu ,0}^4 \right) \Big /\left( \frac{3}{8 \pi } \frac{M_\textrm{Pl}^2 H_{0}^2}{h^2} \right) = 5.70 \times 10^{-6} \, \ \nonumber \\ \Omega ^\mathrm{(nr)}_{\nu } h^2= & {} \left( \frac{3}{2} \frac{\zeta (3)}{\pi ^2} T_{\nu ,0}^3 \, \sum _{i} m_{{\nu }_{i}} \right) \Bigg /\left( \frac{3}{8\pi } \frac{M_\textrm{Pl}^2 H_{0}^2}{h^2} \right) \nonumber \\ {}= & {} \sum _{i} \frac{m_{{\nu }_{i}}}{93.03 \, \textrm{eV}} \, \end{aligned}$$

which reproduces the relic neutrino abundance computed, e.g., in Ref. [65] to the per mil level.

In order to obtain $T_\gamma (t)$ and $T_\nu (t)$ from Eq. (3), we have made use both of Eq. (1) together with Eq. (2). At this point, to complete the study of the thermodynamic background, we must extract the scale factor *a* as a function of time *t* and temperature $T_{\gamma }$. This can be accomplished by applying (again) the notion of local thermodynamic equilibrium, which allows one to introduce the entropy density for each species *i* as: $s_{i} = (\rho _{i} + p_{i} - \mu _{i} \, n_{i})/T_{i}$, where $n_{i}$ is the number density of the species with associated chemical potential $\mu _{i}$.

For negligible chemical potentials, the total entropy density of the Universe $s_{tot}$ per comoving volume must be conserved as a consequence of energy-momentum conservation, Eq. (4). Then, during radiation domination $s_{tot}$ roughly scales as $T_{\gamma }^3$, underlying the approximate relation $a \propto 1/T_{\gamma }$. Nevertheless, even under the assumption of $\mu _{i}/T_{i} \ll 1$, the entropy of each species is generally not separately conserved due to heat exchanges related to the interactions with other species. The Boltzmann equation for $s_{i}$ generally follows (see, e.g., the discussion in Refs. [47, 66]):

7 $$\begin{aligned} \frac{d s_{i}}{dt} + 3 H s_{i} = \frac{\delta C_{i}}{T_{i}} -\frac{\mu _{i}}{T_{i}} \left( \frac{d n_{i}}{dt} + 3 H n_{i} \right) \ , \end{aligned}$$

where the first collision term (divided by the temperature) is the one appearing in the Boltzmann equation for the density $\rho _{i}$, while the second collision term has been rewritten using the Boltzmann equation for the number density $n_{i}$.^4^ In the SM, in the limit^5^$\mu _{e}/T_{\gamma } \ll 1$, we use Eq. (7) for the electromagnetic bath to pin down the relation between *a* and $T_{\gamma }$; with $\bar{s}_\textrm{pl} \equiv (s_{\gamma } + s_{e^{\pm }})/T^3_{\gamma }$, we get:

8 $$\begin{aligned}{} & {} (T_{\gamma }a)^{-3} \ \, \frac{d\left( \bar{s}_\textrm{pl} T_{\gamma }^3 a^3\right) }{d \ln a} = - \frac{\delta C_{\nu }}{H T^4_{\gamma }} \equiv - \mathcal {N}_{\nu }\ \ \Leftrightarrow \nonumber \\ \ \ {}{} & {} a(T_{\gamma }) \ \, = a_{0} \exp \left( - \int _{T_{\gamma ,0}}^{T_{\gamma }} \, \frac{d T}{T} \frac{3 \bar{s}_\textrm{pl} + T \, \bar{s}_\textrm{pl}^{\,\prime }}{3 \bar{s}_\textrm{pl}+ \mathcal {N}_{\nu }}\right) . \end{aligned}$$

Knowing all the thermodynamic quantities as a function of $T_{\gamma }$ in the integrand above, Eq. (8) allows one to extract $a(T_{\gamma })$ up to the scale-factor value of today, $a_{0}$, customarily defined as 1. Note that for $T_{\gamma } \lesssim m_{e}$ one has $\bar{s}_\textrm{pl}^{\,\prime } = 0$, and taking the limit $\mathcal {N}_{\nu } \rightarrow 0$, the expected scaling set by $d(s_{\gamma } a^3)/dt = 0$ is easily recovered. The solution in Eq. (8) precisely tracks the relation between the scale factor and $T_{\gamma }$ in the case of non-instantaneous decoupling of neutrinos. While in the SM these effects are tiny (since $\mathcal {N}_{\nu }/3 \ll \bar{s}_\textrm{pl}$), they could become non-negligible in a BSM scenario.

It is worth noting that given $T_{\gamma }(t)$ from the solution of Eq. (3) and $a(T_{\gamma })$ from Eq. (8), one obtains *a*(*t*) as a byproduct, which allows to assess the evolution of the number density of baryons in *t* or $T_{\gamma }$ during the BBN era, since by definition: $n_{B} \propto 1/a^{3}$.

### Neutron freeze out beyond the born approximation

Shortly after hadrons form, neutrons and protons are non-relativistic species that do not contribute appreciably to the total energy budget stored in the thermal bath. Nevertheless, their abundance is eventually responsible for the tiny fraction of light primordial elements relative to hydrogen which are observable today in pristine astrophysical environments.

According to local thermodynamic equilibrium, the relative number density of nucleons is initially given by the Maxwell-Boltzmann distribution:

9 $$\begin{aligned} \begin{aligned} \left( \frac{n_\textrm{n}}{n_\textrm{p}}\right) \Big |_{T_{\gamma } \, \gg \mathrm{\, MeV}} = \left( \frac{m_\textrm{n}}{m_{\textrm{p}}}\right) ^{3/2}\exp \left( -\frac{\mathcal {Q}}{T_{\gamma }}-\frac{\mu _{\mathcal {Q}}}{T_{\nu }}\right) \, \end{aligned} \end{aligned}$$

where $\mathcal {Q} = m_\textrm{n} -m_\textrm{p}$, $\mu _{\mathcal {Q}} = \mu _\textrm{n}-\mu _\textrm{p}$, $m_\textrm{n,p}$ and $\mu _\textrm{n,p}$ are the mass and chemical potential of neutrons and protons. For clarity, we have used $T_{\nu } = T_{\gamma }$ (valid for temperatures well above MeV) in the $\mathcal {Q}$ term, but retain $T_{\nu }$ explicitly in the $\mu _{\mathcal {Q}}$ term. Assuming $\mu _\textrm{n} \simeq \mu _\textrm{p}$ (e.g. a negligible contribution from lepton chemical potentials), Eq. (9) implies that at equilibrium $n_\textrm{n} \simeq n_\textrm{p}$. Indeed, fast electroweak processes efficiently convert $n \leftrightarrow p$, i.e. both:

$$\begin{aligned} \Gamma _{\textrm{n} \, \rightarrow \, \textrm{p}}\equiv & {} \Gamma ({n \, e^{+} \,\rightarrow \, p \, \bar{\nu }}) + \Gamma (n \, \bar{\nu } \, \rightarrow \, p \, e^{-}) \\{} & {} + \Gamma (n \, \rightarrow \, p \, e^{-} \, \bar{\nu }), \\ \Gamma _{\textrm{p} \, \rightarrow \, \textrm{n}}\equiv & {} \Gamma (p \, e^{-} \,\rightarrow \, n \, \bar{\nu }) + \Gamma (p \, \bar{\nu } \, \rightarrow \, n \, e^{+})\\ {}{} & {} + \Gamma (p \, e^{-} \, \bar{\nu } \, \rightarrow \, n), \end{aligned}$$

are $\gg H$ and govern the Boltzmann equations for the nucleon yields $Y_\textrm{n,p} \equiv n_\textrm{n,p} / n_{B} = n_\textrm{n,p} / (n_\textrm{n}+n_\textrm{p})$:

10 $$\begin{aligned} \frac{d Y_{\textrm{n}}}{dt}= & {} \Gamma _{\textrm{p} \, \rightarrow \, \textrm{n}} \, Y_{\textrm{p}} - \Gamma _{\textrm{n} \, \rightarrow \, \textrm{p}} \, Y_\textrm{n} \, \nonumber \\ \frac{d Y_{\textrm{p}}}{dt}= & {} \Gamma _{\textrm{n} \, \rightarrow \, \textrm{p}} \, Y_{\textrm{n}} - \Gamma _{\textrm{p} \, \rightarrow \, \textrm{n}} \, Y_{\textrm{p}} \, \end{aligned}$$

that at equilibrium are: $Y_{\textrm{n}} = 1-Y_{\textrm{p}} = \Gamma _{\textrm{p} \, \rightarrow \, \textrm{n}}/(\Gamma _{\textrm{p} \, \rightarrow \, \textrm{n}} + \Gamma _{\textrm{n} \, \rightarrow \,\textrm{p}}) \simeq 1/2$, in agreement with Eq. (9). These reactions guarantee chemical equilibrium among the involved species, implying $\mu _{\mathcal {Q}} \simeq -\mu _{\nu }$. Equation (9) thus demonstrates that a primordial non-zero lepton asymmetry in the neutrino sector [67, 68] can impact the initial conditions for BBN by altering the neutron-to-proton ratio, with notable cosmological consequences [35, 69].

At temperatures close to neutrino decoupling, $n \leftrightarrow p$ conversion falls out of equilibrium, freezing out the neutron-to-proton ratio to $\sim 1/6$ (in the SM), up to finite neutron lifetime effects [59, 60]. The weak rates for neutron freeze out require the evaluation of an involved multi-dimensional phase-space integral: e.g. for $n \, e^{+} \rightarrow p \, \bar{\nu }$ (and similarly for the others) [70]:

11 $$\begin{aligned}{} & {} Y_{\textrm{n}} \, \ \, \Gamma ({n \, e^{+} \,\rightarrow \, p \, \bar{\nu }}) = \frac{16 \pi ^4}{n_{B}} \int d \Pi _\textrm{n} d \Pi _{e} d \Pi _\textrm{p} d \Pi _{\nu } \, |\mathcal {M}|^2 \, \nonumber \\{} & {} \quad \times \delta ^{(4)}(P_{\textrm{n}}+P_{e}-P_{\textrm{p}}-P_{\nu }) \, f_{\textrm{n}} f_{e} (1-f_{\textrm{p}})(1-f_{\nu }), \end{aligned}$$

where $d \Pi _{i}$ and $P_{i}$ are the Lorentz-invariant phase-space element and 4-momentum of the particle *i*, $f_{i}$ is the relativistic thermal distribution of the species *i* in the rest frame of the thermal bath, and $\mathcal {M}$ is the full matrix element of the process summed over initial and final spins. The latter can be computed from the weak effective theory for $\beta$ decay [71]:

12 $$\begin{aligned} \mathcal {L}_{\textrm{F}}= & {} - \frac{2G_{\textrm{F}}}{\sqrt{2}} \, V_\textrm{ud} \, \, \bar{\nu }(x) \, \gamma _{\mu }\,e_{L}(x) \, \Big \{\,\bar{n}(x) \gamma ^{\mu }(1 - g_\textrm{A} \, \gamma _{5})p(x) \nonumber \\{} & {} + \frac{\kappa }{2 m_\textrm{N}} \partial _\nu \left[ \bar{n}(x) \, \sigma ^{\mu \nu } \, p(x) \right] \,\Big \} + h.c., \end{aligned}$$

where $G_{\textrm{F}}$ is the Fermi constant [42], $V_\textrm{ud}$ corresponds to the Cabibbo angle [72], $g_\textrm{A}$ and $\kappa$ are the axial-current and weak-magnetism constant of the nucleon of mass $m_\textrm{N}$ [73], and $\sigma _{\mu \nu } \equiv i \, (\gamma _{\mu }\gamma _{\nu }-\gamma _{\nu }\gamma _{\mu })/2$. The computation of $|\mathcal {M}|^2$ can be found in detail in Appendix B of Ref. [47] (see also [70, 74]).

While expressions like Eq. (11) can be reduced to a five-dimensional integral in phase space by exploiting the symmetries of the problem, a dramatic simplification is obtained in the limit of infinite nucleon-mass at fixed $\mathcal {Q}$ [70, 74]. This is the so-called Born approximation, in which the kinetic energy of the ‘infinitely’ heavy neutrons and protons may be neglected, leading to the simplification: $|\mathcal {M}|^2= 32 \, G_{\textrm{F}}^2V_\textrm{ud}^2(1+3g_\textrm{A}^2)E_e E_\nu E_\textrm{p} E_\textrm{n}\,$. In that limit the $n \leftrightarrow p$ rates read:

13 $$\begin{aligned} \Gamma _\mathrm{n \rightarrow p}^{\infty }= & {} \widetilde{G}_{\textrm{F}}^2 \int _{0}^{\infty } dE_e \,E_e \, \sqrt{E_e^2-m_e^2} \, (E_\nu ^-)^2 \nonumber \\{} & {} \times \left[ f_{\nu }(E_\nu ^-)f_{e}(-E_e)+ f_{\nu }(-E_\nu ^-)f_{e}(E_e)\right] \, \nonumber \\ \Gamma _\mathrm{p \rightarrow n}^{\infty }= & {} \widetilde{G}_{\textrm{F}}^2 \int _{0}^{\infty } dE_e \,E_e \, \sqrt{E_e^2-m_e^2} \, (E_\nu ^+)^2 \nonumber \\{} & {} \times \left[ f_{\nu }(E_\nu ^+)f_{e}(-E_e)+ f_{\nu }(-E_\nu ^+)f_{e}(E_e)\right] \, \end{aligned}$$

where $\widetilde{G}_{\textrm{F}} \equiv G_{\textrm{F}}V_\textrm{ud}\sqrt{(1+3 g_\textrm{A}^2)/(2\pi ^3)}$ and $E_\nu ^\pm = E_{e} \pm \mathcal {Q}$. The outcome of Eq. (13) are rates that generally depend on both background temperatures and chemical potentials (i.e. $T_{\gamma },T_{\nu }$ and $\mu _{\nu }$). For $T_{\nu } = T_{\gamma }$ (and negligible chemical potentials) detailed balance follows as:

14 $$\begin{aligned} \Gamma _\mathrm{p \rightarrow n}^{\infty }/\Gamma _\mathrm{n \rightarrow p}^{\infty } = \exp (-\mathcal {Q}/T_{\gamma }). \end{aligned}$$

The dimensionful factor $\widetilde{G}_{\textrm{F}}$ depends on $V_\textrm{ud}$, $g_\textrm{A}$, and $G_{\textrm{F}}$, whose value is precisely determined by the muon lifetime. However, this factor is often more conveniently extracted from neutron decay in the vacuum, since in the SM:

15 $$\begin{aligned} \tau _\textrm{n}^{-1} = \widetilde{G}_{\textrm{F}}^2 \, m_{e}^5 \, \mathcal {F}_\textrm{n} \ , \end{aligned}$$

where $\mathcal {F}_\textrm{n}$ incorporates a phase-space statistical factor for the neutron decay at zero temperature [75] plus electroweak radiative corrections [76]. For a precise calculation of $\mathcal {F}_\textrm{n}$, see the very recent reassessment in Ref. [77] and references therein. This approach allows one to trade the combination $V_{ud}^2(1+3g_A^2)$ for the measured $\tau _{n}.$^6^ Using Eq. (15), in PRyMordial one can choose to adopt either a normalization of the weak rates based on the determination of the neutron lifetime, or one involving the knowledge of the modified Fermi constant $\widetilde{G}_{\textrm{F}}$.

In the SM the Born approximation predicts a neutron freeze-out temperature of slightly below 1 MeV. At smaller temperatures, the neutron-to-proton ratio is still affected by $\beta$ decay until the Universe cools down sufficiently enough to preclude photo-dissociation of deuterium: for a binding energy $B_\textrm{D} = 2.2$ MeV, this happens at temperatures around

$B_\textrm{D}/\log (1/\eta _{B})\sim \,$0.1 MeV [59, 60]. At that point, virtually all of the neutrons experience two-body nuclear reactions, ultimately resulting in their binding in helium-4, the most stable light element. As a result, the uncertainty on the Born-level theory prediction for helium-4 is only a few % (see Table 5 in [47]).

That said, the present percent-level inference of primordial helium-4 and deuterium [42] and the sub-percent target of future observational campaigns [39] demand the following refinements to Eq. (13):

- QED radiative corrections (in the vacuum) to the $n \leftrightarrow p$ amplitudes of order $\mathcal {O}(\alpha _\textrm{em})$ via virtual- and real-photon emission [82–85] must be computed;
- Finite nucleon-mass effects and non-zero weak magnetism, which induce relative shifts in the weak rates of $\Delta \Gamma /\Gamma \sim \mathcal {O}(10^{-2})$  [70, 74], must be taken into account;
- Finite-temperature effects [84, 86] must be evaluated for sub-percent accuracy.

PRyMordial implements all of these corrections, following the treatment in PRIMAT (see Appendix B of [47]), where particular care was taken to attempt to combine several existing state-of-the-art recipes for electroweak rates beyond the Born approximation.

It is worth noticing that in the context of the SM, the corrections to the Born rates due to the incomplete neutrino decoupling are only marginal [87, 88]. Nevertheless, NP could dramatically alter $T_{\nu }(T_{\gamma })$, $a(T_{\gamma })$ and *a*(*t*), and the departure from the standard value for the weak rates can impact the final BBN abundances in a non-negligible way [31, 33]. As a result, the approach undertaken in Sect. 2.1 is particularly useful not only for the study of neutrino decoupling, but also for a careful assessment of the neutron-to-proton ratio in BSM scenarios.

### Thermonuclear reactions

Local thermodynamic equilibrium implies that at temperatures above neutron decoupling, a nuclear species *i* of atomic number $Z_{i}$, mass number $A_{i}$, spin $s_{i}$, and binding energy $B_{i}$ follows a Boltzmann distribution with internal degrees of freedom: $g_{i} = 2 s_{i}+1$; mass: $m_{i} = Z_{i} m_\textrm{p} + (A_{i}-Z_{i}) m_\textrm{n }- B_{i}\,$; and chemical potential: $\mu _{i}= Z_{i} \mu _\textrm{p} + (A_{i}-Z_{i}) \mu _\textrm{n }$. In terms of the yield $Y_{i} \equiv n_{i}/n_{B}$, this equilibrium distribution reads:

16 $$\begin{aligned} Y_{i}\big |_{T_{\gamma }\gtrsim \textrm{MeV}}= & {} g_{i} \, 2^{(3 A_{i}-5)/2} \, Y_\textrm{p}^{Z_i} Y_{\textrm{n}}^{A_i-Z_i} \exp \left( \frac{B_{i}}{T_{\gamma }}\right) \nonumber \\{} & {} \times \left( \frac{\zeta (3)\, \eta _{B}}{\sqrt{\pi }}\right) ^{A_{i}-1} \left( \frac{m_{i} \, T_{\gamma }^{A_{i}-1}}{m_\textrm{p}^{Z_i} m_\textrm{n}^{A_i-Z_i}} \right) ^{3/2}, \end{aligned}$$

where we made use of: $n_{B}/\eta _{B} = 3 \, \zeta (3)\,T^3_{\gamma }/(2\pi ^2)$. This expression holds for the nucleons ($A_\textrm{N} = 1$, $B_\textrm{N} = 0$) themselves, and is consistent with Eq. (9). Importantly, it offers another handle on the estimate for the start of nucleosynthesis as the time in which the relative abundance of neutrons after freeze out becomes comparable to deuterium as dictated by Eq. (16), and pointing again to a temperature of about 0.1 MeV.

Starting from the initial conditions, abundances are determined by a network of Boltzmann equations that generalize Eq. (10) (see, e.g., Refs.  [89, 90]) to include the relevant nuclei:

17 $$\begin{aligned} \frac{dY_{i}}{dt}= & {} \sum _{} \mathcal {S}^{(R)}_{i} \left[ \Gamma ^{(R)}_{\dots \, \rightarrow \, i \, \dots } \times \prod _{j} \left( \frac{Y_{j}^{\mathcal {S}^{(R)}_{j}}}{\mathcal {S}^{(R)}_{j}!} \right) \right. \nonumber \\ {} & {} \left. - \Gamma ^{(R)}_{i \, \dots \rightarrow \, \dots } \times \prod _{k} \left( \frac{Y_{k}^{\mathcal {S}^{(R)}_{k}}}{\mathcal {S}^{(R)}_{k}!} \right) \right] , \end{aligned}$$

where the sum *R* is performed over all reactions involving the nuclear species *i*; $\mathcal {S}^{(R)}_{i}$ is the stoichiometric coefficient $\mathcal {S}$ for the species *i* in the nuclear reaction *R*; and the products *j* and *k* run over all of the initial and final states of the reaction with (thermo)nuclear rate $\Gamma ^{(R)}_{ \dots \rightarrow i \, \dots }$ or $\Gamma ^{(R)}_{i \, \dots \rightarrow \dots }$.

Given the range of energies characterizing the BBN era, the nuclear reaction rates of interest can be measured in the laboratory, and are often tabulated as [91]

$$\begin{aligned} \widetilde{\Gamma }_{ i \dots l\rightarrow j \dots m} \equiv N_{A}^{\mathcal {S}_{i} \dots \mathcal {S}_{l}-1} \langle \sigma _{ i \dots l\rightarrow j \dots m} \, v \rangle \ \ , \end{aligned}$$

where $N_{A}$ is Avogadro’s number (typically expressed in units of mol$^{-1}$), and the velocity averaged cross section is obtained by weighting the appropriate cross section by the Maxwell-Boltzmann velocity distribution for the non-relativistic species (see e.g. Ref. [92] for a detailed description). By definition, for a given number-density rate $\langle \sigma ^{(R)}_{i \dots \rightarrow \dots } v \rangle$, the corresponding abundance rate $\Gamma ^{(R)}_{i \dots \rightarrow \dots }$ is:

18 $$\begin{aligned} \Gamma _{ i \dots l\rightarrow j \dots m}= & {} n_{B}^{\mathcal {S}_{i} \dots \mathcal {S}_{l}-1} \langle \sigma _{i \dots l\rightarrow j \dots m} \, v \rangle \nonumber \\&=\ {}&(n_{B}/N_{A})^{\mathcal {S}_{i} \dots \mathcal {S}_{l}-1} \, \widetilde{\Gamma }_{ i \dots l\rightarrow j \dots m}. \end{aligned}$$

A priori, Eq. (17) includes the rates of both forward and reverse reactions in the evolution of the abundance of the nuclear species *i*. Nevertheless detailed balance implies for $T_{\gamma }\gtrsim \textrm{MeV}$:

19 $$\begin{aligned} \left( \frac{Y_{j}^{\mathcal {S}_{j}} \dots Y_{m}^{\mathcal {S}_{m}}}{Y_{i}^{\mathcal {S}_{i}} \dots Y_{l}^{\mathcal {S}_{l}}}\right) = \ \frac{\langle \sigma _{i \dots l\rightarrow j \dots m} v \rangle /(\mathcal {S}_{i}! \dots \mathcal {S}_{l}!)}{\langle \sigma _{j \dots m\rightarrow i \dots l} v \rangle /(\mathcal {S}_{j}! \dots \mathcal {S}_{m}!)}, \end{aligned}$$

since local thermodynamic equilibrium ensures that the forward and reverse reactions should balance. Thus, it is easy to evaluate the reverse reaction rates given the forward ones. It is customary to parameterize the relationship as:

20 $$\begin{aligned} \frac{\langle \sigma _{j \dots m\rightarrow i \dots l} v \rangle }{\langle \sigma _{i \dots l\rightarrow j \dots m} v \rangle } = \alpha _R \ T_{9}^{\beta _R} \,\exp (\gamma _R / T_{9}) \, \ , \end{aligned}$$

with $T_{9} \equiv T_{\gamma }/ (10^9 \, \text {K})$ and where the constants $\alpha _R$, $\beta _R$, and $\gamma _R$ for a given process *R* from e.g. the up-to-date nuclear database of Ref. [93] via Eq. (16).

PRyMordial solves the general system of equations Eq. (17) following the strategy of Ref. [47] which conveniently breaks nucleosynthesis into three steps:

1. We analyze $n \leftrightarrow p$ conversion by solving Eq. (10) from an initial temperature of $\mathcal {O}(10)$ MeV (and initial conditions from Eq. (9)) down to standard neutron freeze out, around MeV;
2. We use the values of $Y_\textrm{n,p}$ obtained from (1) together with Eq. (16) and evolve with a network comprised of the 18 key thermonuclear rates for the abundance of *n*, *p* together with all of the nuclides up to $A=8$ and $Z=5$^7^ down to the temperature where deuterium photo-dissociation becomes inefficient, around 0.1 MeV;
3. We further evolve the network with the full set of thermonuclear processes and with initial conditions given by the nuclide yields obtained in step (2), evolving the abundances of the aforementioned nuclides down to $\mathcal {O}$(keV) (i.e., well below $e^{\pm }$ annihilation), when BBN is over.

The output of Step (3) is the abundances of the light-element originating from BBN. To compare with data, it is customary to quote helium-4 in terms of the primordial mass fraction^8^

21 $$\begin{aligned} Y_{P} \equiv 4 \times Y_{^4 \mathrm He} \simeq \rho _{^4 \mathrm He}/\rho _{B} \, . \end{aligned}$$

The other primordial elements under the lamppost of astrophysical observations are deuterium, helium-3 and lithium-7 (see, e.g., [11] for a recent report on the status of these measurements), which are usually quoted in terms of the relative number densities with respect to hydrogen:

22 $$\begin{aligned} i / \textrm{H} \equiv Y_{i}/Y_{ \mathrm p} = n_{i}/n_{ \mathrm H} \ \ , \ \textrm{where}~i = \textrm{D},\, ^3 \textrm{He},\, ^7 \textrm{Li} \, . \end{aligned}$$

Notice that the final yield of primordial helium-3 receives a contribution from unstable species such as tritium; likewise, the final amount of lithium-7 includes the decay of beryllium-7.

The literature contains several publicly accessible compilations of the thermonuclear rates relevant for BBN. It is important to note that there are several different parameterizations of these rates adopted in BBN studies, and they differ not only with respect to the theoretical approach, but also with respect to the measured nuclear reaction data included in fitting them. To highlight a few of the more important approaches:

- The NACRE II database [95] collects an extended evaluation of reaction rates of charged-particle induced reactions on target nuclides with mass number $A<16$, adopting the so-called potential model [91] to describe nuclear cross sections in the energy range of interest.
- PRIMAT tabulates an extensive catalogue (comprising more than 400 reactions), characterized by several nuclear cross sections evaluated via refined statistical analyses within *R*-matrix theory [96–99] or computed using dedicated numerical tools, e.g., the TALYS code [100].
- PArthENoPE implements semi-analytic expressions resulting from polynomial fits to nuclear data including theory modeling of screening and thermal effects [92, 101]; data-oriented analyses relevant for BBN rates can be also found in Refs. [102, 103].

**Fig. 1:**
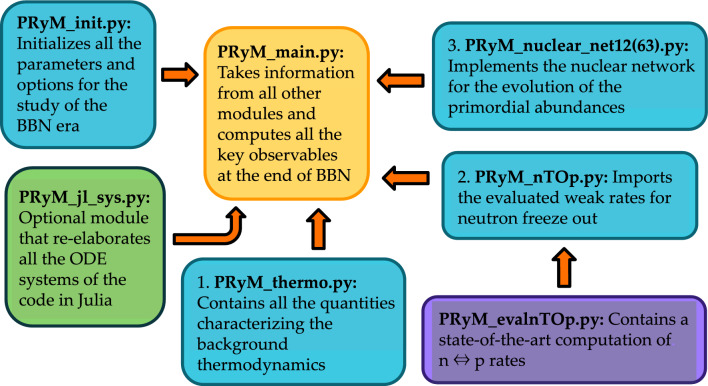
PRyMordial in a nutshell: Schematic of the modules making it up and their inter-relations

If one limits the scope to precise predictions of the helium-4 and deuterium abundances, the relevant portion of the nuclear network simplifies considerably, contracting to $\mathcal {O}(10)$ processes [104]. Thus, PRyMordial offers the option of restricting the BBN analysis to a small network of 12 key reactions [105], implemented according to two different sets of thermonuclear rates: the first is largely based on the NACRE II compilation, whereas the second is based on the tabulated rates in PRIMAT. These two sets differ marginally in their predictions for helium-4, but lead to relevant differences in the prediction for deuterium, as discussed at length in Ref. [54], after the important measurement carried out by the LUNA collaboration [106].^9^ For the most precise prediction of lithium-7, PRyMordial offers the possibility to solve a nuclear network including the 51 additional reactions listed in Appendix B, by adopting part of the network in Ref. [100] included in the PRIMAT database.

PRyMordial handles uncertainties on the tabulated thermonuclear rates $\widetilde{\Gamma }^{(R)}$ by providing (for each forward^10^ nuclear reaction) a set of median values, $\langle \widetilde{\Gamma }^{(R)} \rangle$ together with an uncertainty factor $\Delta \widetilde{\Gamma }^{(R)}$, corresponding to a sample of temperatures. Following the method outlined in Refs. [107, 108], to perform a MC analysis with PRyMordial one should treat the provided thermonuclear rates as log-normal distributed, implying that for each nuclear process *R* a random realization of the thermonuclear rate will be:

23 $$\begin{aligned} \log \widetilde{\Gamma }^{(R)} = \log \,\langle \widetilde{\Gamma }^{(R)} \rangle + p^{(R)} \log \Delta \widetilde{\Gamma }^{(R)} \ , \end{aligned}$$

where $p^{(R)}$ is a temperature-independent coefficient following a normal distribution [109]. Hence, in order to properly take into account the uncertainties of the thermonuclear rates in a MC analysis of BBN, one should independently vary the nuisance parameters $p^{(R)}$ for all the reactions *R* included in the study, see, e.g., the work carried out in Ref. [35] and the MC examples presented in Sect. 3.

## How to use PRyMordial

In this section we provide some example code that demonstrates the use of PRyMordial. We start by detailing the modules of the code including their inputs and key parameters. We show how to implement a state-of-the-art analysis of the BBN era within the SM. Finally, we provide a concise description on how to use the code for the study of NP, and discuss how to implement and analyze generic BSM scenarios.

### Structure of the code and Hello, World!

PRyMordial is a numerical tool dedicated to efficiently and accurately evaluate in the SM and beyond all the key observables related to the BBN era, discussed in Sect. 2, namely:

- The number of effective relativistic degrees of freedom, $N_\textrm{eff}$, Eq. (5) ;
- The cosmic neutrino abundance today, $\Omega _{\nu } h^2$, Eq. (6) ;
- The helium-4 mass fraction (both for BBN and CMB), $Y_{P}$, Eq. (21) ;
- The relative number density of deuterium, helium-3 and lithium-7, Eq. (22) .

In contrast to other BBN codes available, PRyMordial begins by computing the thermal background from first principles. As a byproduct of the determination of $N_\textrm{eff}$ and $\Omega _{\nu }h^2$, the relationship between time, scale factor and temperature of relativistic species is determined precisely, including effects from non-instantaneous decoupling within and beyond the Standard Model.

Next, PRyMordial evaluates the weak rates for neutron freeze out via a state-of-the-art implementation that includes nucleon finite-mass effects, one-loop QED corrections and finite-temperature effects. While the latter are typically negligible within current observational precision and can be conveniently stored between runs, the remainder are generally recomputed for each iteration of a generic BBN analysis.

Finally, PRyMordial solves a network of nuclide reactions for their yields within three different physical regimes: (i) a high-temperature era in which one can restrict the study to nucleons with an initial temperature of $\mathcal {O}(10)~$ MeV and a final temperature close to neutrino decoupling; (ii) a mid-temperature era from $\mathcal {O}(1)~$ MeV down to $\mathcal {O}(0.1)~$ MeV, during which photo -dissociation of nuclear bound states is relevant; (iii) and a low temperature era starting at $\mathcal {O}(0.1)~$MeV during which PRyMordial follows all of the nuclear species of interest, which ends at a temperature well below $e^{\pm }$ heating of the thermal bath, i.e. down to $\mathcal {O}(1)~$keV. Local thermal equilibrium sets the initial nuclide abundances and detailed balance determines all of the reverse reactions included in the chosen set of nuclear reactions. These three regimes are matched such that the solution for each one provides the initial conditions for the successive period.

PRyMordial is a Python package with optional dependencies which allow more advanced users to speed up execution by exploiting the Julia programming language. The recommended libraries and general requirements are tabulated in Appendix A. As highlighted in Fig. 1, PRyMordial is organized in five primary modules:

- PRyM_init.py is an initialization module where physical constants and Boolean flags for user-controlled options are defined; in particular, three main blocks for input parameters are found:
  - $\star$ Fundamental constants, masses (in natural units), initialized according to the PDG [42]^11^
  - $\star$ Additional parameters needed for the evaluation of the $n \leftrightarrow p$ rates beyond the Born level;
  - $\star$ Cosmological inputs including the CMB temperature and the abundance of baryonic matter [24].
  Boolean flags allow the user to switch on/off the following options:
  - $\circ$ verbose_flag: Allows the user to run the code with all of the internal messages enabled;
  - $\circ$ numba_flag: If True, speeds up some numerical integrations, if the Numba library is installed;
  - $\circ$ numdiff_flag: If True, performs numerical derivatives using Numdifftools library;
  - $\circ$ aTid_flag: Controls the inclusion of incomplete-decoupling effects in the determination of the scale factor as a function of time and temperature;
  - $\circ$ compute_bckg_flag: If True, recomputes the thermodynamic background as presented in Sect. 2.1 (via save_bckg_flag the outcome can be stored in a file for future runs);
  - $\circ$ NP_thermo_flag: If True, includes the contribution(s) of new (interacting) species to the dynamics of the thermal bath (by default, one must also provide a NP temperature);
  - $\circ$ NP_nu_flag: If True, includes new species thermalized with the neutrino bath;
  - $\circ$ NP_e_flag: If True, includes new species thermalized with the plasma;
  - $\circ$ compute_nTOp_flag: If True, recomputes weak rates beyond Born as discussed in Sect. 2.2 (via save_nTOp_flag the outcome can be stored in a file for future runs);
  - $\circ$ nTOpBorn_flag: If True, adopts the Born approximation for the neutron freeze out;
  - $\circ$ compute_nTOp_thermal_flag: If True, recomputes thermal corrections to $n \leftrightarrow p$ rates via Vegas (since this is numerically intensive, we recommend save_nTOp_thermal_flag = True);
  - $\circ$ tau_n_flag: If True, uses the neutron lifetime to normalize the weak rates, see Sect. 2.2;
  - $\circ$ NP_nTOp_flag: If True, includes NP affecting $n \leftrightarrow p$ rates in units of the Born rates;
  - $\circ$ smallnet_flag: If True, restricts the nuclear network to the set of 12 key nuclear processes collected in Table 1 of Appendix B;
  - $\circ$ nacreii_flag: If True, the key nuclear rates adopted in PRyMordial will be mostly based on NACRE II compilation rather than those of PRIMAT, see Sect. 2.3;
  - $\circ$ NP_nuclear_flag: If True, shifts the nuclear rates due to NP in units of the standard ones;
  - $\circ$ julia_flag: If True, solves all of the systems of ordinary differential equations using routines in the SciML kit [58] developed for the Julia programming language; the optional dependencies described in Appendix A are then required.
  This module also loads the tabulated nuclear rates (as well as the coefficients of Eq. (20)).
- PRyM_thermo.py is the module where all of the thermodynamic quantities for the species contributing to the expansion of the Universe during radiation domination are defined, together with all the collision terms that enter in Eq. (3) and Eq. (7).
- PRyM_nTOp.py is the module which imports the weak rates for $n \leftrightarrow p$ conversion described in Sect. 2.2, either relying on the additional module PRyM_evalnTOp.py – where the actual computation of the rates is performed from scratch – or by loading pre-stored rates from a file.
- PRyM_nuclear_net12.py and PRyM_nuclear_net63.py are the modules which set up the systems of ordinary differential equations – see Eq. (17) – involving the nuclear rates loaded by PRyM_init.py. The Boolean flag smallnet_flag controls whether PRyMordial sets up and solves the smaller network of 12 key reactions or the full set of 63 nuclear processes.
- PRyM_main.py is the main module, which calls the other modules to solve for the thermodynamic background, compute $N_{\textrm{eff}}$ and the cosmic neutrino abundance, and solve for the nuclide yields. It contains the Python class PRyMclass(), designated to return all the cosmological observables in the package.

- PRyM_jl_sys.py is an optional module which allows the user to solve all of the systems of differential equations in PRyM_main.py by taking advantage of the numerically efficient routines that are part of the SciML kit [58] developed in Julia. In some cases, this significantly speed up the execution time of the code (to a degree depending on both the adopted precision of the computation as well as the specific choice of differential-equation solver).

After downloading PRyMordial, the code can be used immediately. To run a Hello, World!-style example, the user would enter the package folder, start an interactive Python session, and type: which executes a BBN computation and fills the array res with the values of:

$$\begin{aligned} {[}~N_\textrm{eff}, \, \Omega _{\nu }h^2 \times 10^6~\mathrm{(rel)}, \, \sum m_{\nu }/\Omega _{\nu }h^2 [\textrm{eV}], Y_P^\mathrm{(CMB)}, \\ Y_P^\mathrm{(BBN)}, \, D/H \times 10^5,\, ^3\mathrm{He/H} \times 10^5,\, ^7\mathrm{Li/H} \times 10^{10}~ ]. \end{aligned}$$

Located in the same folder are:

- a folder PRyM in which all of the modules described above reside;
- a folder PRyMrates in which all the essential thermal, weak and nuclear rates are present, and where new evaluations of them can be stored;
- a script named runPryM_julia.py that provides a simple example for the user as to how to use the package, with execution-time benchmarking in both standard and Julia modes.

**Fig. 2:**
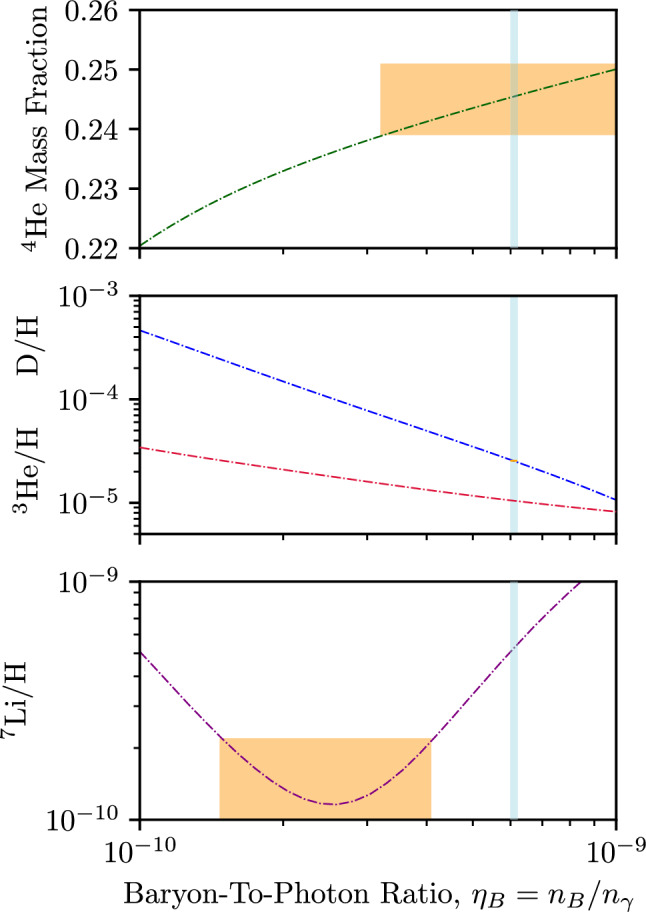
Primordial abundances of helium-4, deuterium, helium-3, and lithium-7 as predicted by PRyMordial within the SM, as a function of the cosmic baryon density. Central predictions are shown without theory uncertainties (i.e. using the nominal nuclear rates for the largest set implemented in the package with the NACRE II compilation for the key processes) and at the central values of all of the inputs. Measurements of light-element abundances (orange) as well as the CMB constraint on the baryon-to-photon ratio (cyan) follow from Figure 24.1 of the PDG [42]

In the following subsections we present more sophisticated examples illustrating PRyMordial’s capabilities.

### Standard model examples: the PDG plot and Monte Carlo analysis

In an interactive session in Python, any default value in PRyM_init.py can be changed using the syntax: This includes the Boolean flags listed in the previous subsection. Hence – to perform a run with: (i) the computation of the thermal background from scratch, including non-instantaneous decoupling effects; (ii) the ab-initio evaluation of the weak rates for neutron freeze out; and (iii) the inclusion of key nuclear processes based on the tabulated rates of the NACRE II compilation – one should type: The array res is assigned the same values as in the Hello, World! example, above. This code also stores the results for the thermal background and $n \leftrightarrow p$ rates for future runs. Consequently, a subsequent call with the same setup can be made faster: While it may be necessary in general to recompute the thermal background and/or the rates for neutron freeze out, there are cases for which storing the outcome of these computations can be computationally advantageous. An example is the classic PDG review BBN plot of the primordial abundances as a function of the baryon-to-photon ratio $\eta _{B}$ [42]. Once thermal background and weak rates have been stored, the behaviour of the abundances in the PDG Figure 24.1 can be reproduced with PRyMordial: The outcome of this code is illustrated in Fig. 2, which adopts the largest nuclear network for the most accurate prediction of the relative abundance of lithium-7. It is worth noting that the BBN prediction for deuterium matches observations of quasar absorption systems, and is also in line with the cosmological abundance of baryons independently determined from the CMB (without a BBN prior). As pointed out in Ref. [54] and further scrutinized in Ref. [35], this test of concordance would fail if the PRIMAT rates were to be adopted, i.e. nacreii_flag = False.

**Fig. 3:**
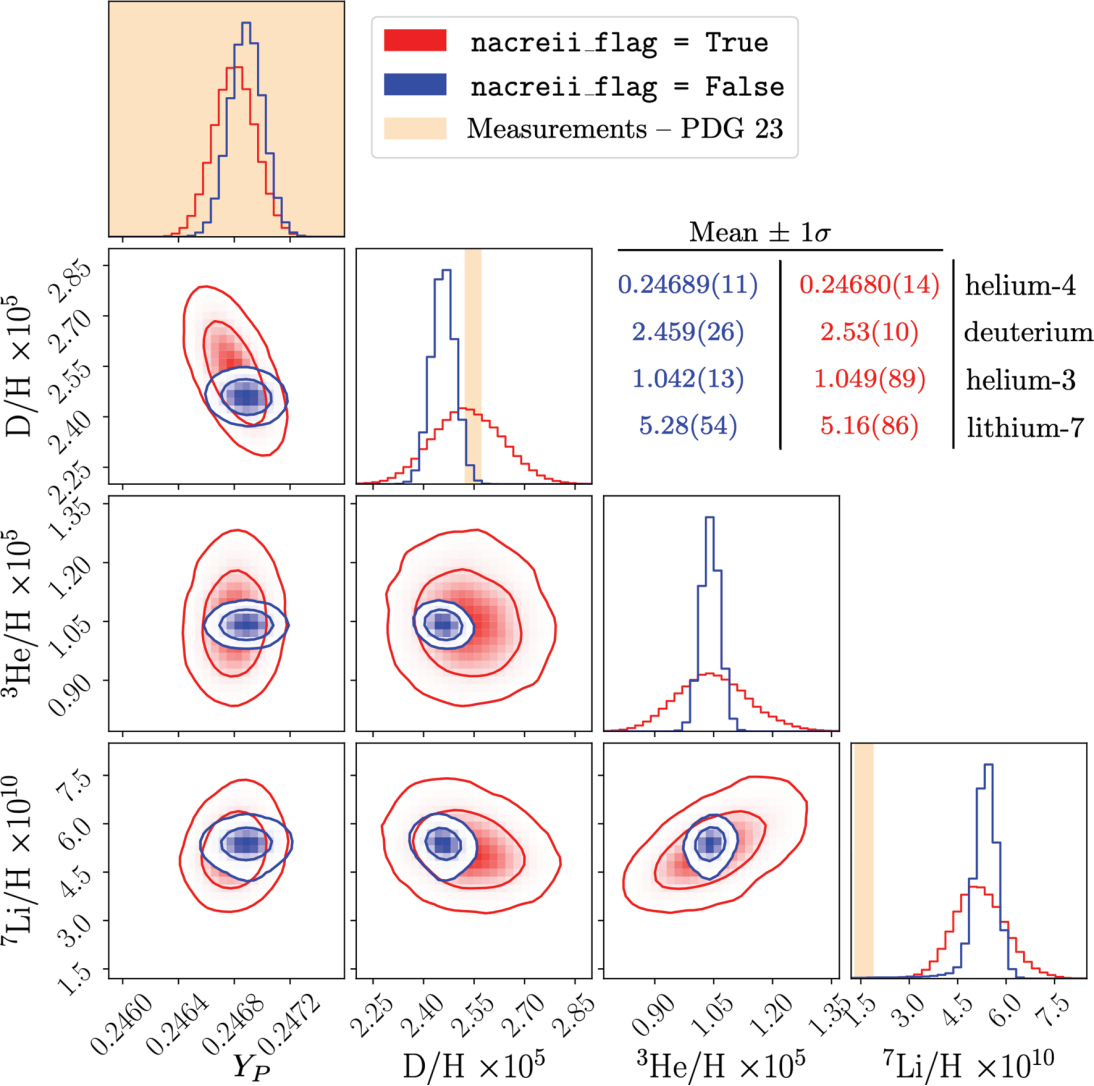
1D probability distributions (and 2D joint 68% and 95% probability regions) for the light primordial abundances predicted in the SM with PRyMordial. Predictions are obtained using a Gaussian prior for the neutron lifetime $\tau _{n} = 878.4 \pm 0.5$ s (comprising the eight best measurements from ultra-cold neutron experiments combined in Ref. [42]), and the cosmic baryon density, $\Omega _{B}h^2 = 0.02230 \pm 0.00020$ (from Table 5 of Ref. [24] for the analysis with an uninformative $Y_{P}$ prior). The large network of nuclear reactions has been used, implying an additional 63 nuisance parameters varied with a log-normal distribution. Two different sets of key nuclear rates have been considered on the basis of the Boolean flag nacreii_flag, and the statistics of the marginalized distributions for each case is presented

To perform a Monte Carlo analysis of the SM predictions taking into account uncertainties (similar to the one presented in Ref. [35]):

The output maps out the probability distributions, shown in Fig. 3, where the light elements at the end of the BBN era are predicted within the SM via a MC analysis that involves: (i) a cosmological prior on the cosmic baryon abundance; (ii) a particle-physics measurement prior on the neutron lifetime; and (iii) a dedicated treatment of the uncertainties in the rates of the nuclear processes. Figure 3 displays the “deuterium anomaly” present for the PRIMAT compilation of the key nuclear rates, and further shows that it is completely washed out when one employs the NACRE II database.^12^

Figure 3 suggests that the “primordial lithium problem” stands out as statistically significant, regardless of the approach undertaken for the nuclear network. However, the up-to-date analysis of the lithium problem in Ref. [94] points out that the predicted primordial abundance of lithium-7 could be depleted via stellar (and cosmic-ray) nucleosynthesis. Given this argument, the observational inference of Figs. 2 and 3, in which the observations lie below the theoretical prediction for primordial lithium-7, are consistent with a resolution for this long-standing puzzle.

### New physics examples: new interacting sectors and big bang nucleosynthesis

PRyMordial allows the user to perform state-of-the-art analyses for Physics beyond the SM in the Early Universe. A few options already built-in to the current release include:

- additional relativistic degrees of freedom contributing to the expansion rate of the Universe in the form of a shift of $N_\textrm{eff}$, see Eq. (5);
- a non-zero chemical potential for neutrinos, influencing both the cosmological expansion rate as well as the equilibrium distributions in the weak processes for neutron-to-proton conversion;
- Boolean flags specific to the study of new species interacting with the plasma and/or neutrino bath, as well as flags implementing a new entire sector with temperature $T_\textrm{NP} \ne T_{\gamma ,\nu }\,$;
- a Boolean flag and a dedicated parameter encoding NP effects as a phenomenological modification of $n \leftrightarrow p$ conversion rates (in units of the Born rates);
- a set of parameters that allow one to similarly investigate NP effects in the nuclear processes as a simple shift in terms of the median rate of each process.

The first two have been extensively investigated in Ref. [35], and thus we focus here on the others. The following is code demonstrating how to implement an electrophilic species in thermal equilibrium with the SM at BBN: One can similarly evaluate a thermalized neutrinophilic species by replacing the Boolean flag at the top of the script with: PRyMini.NP_nu_flag = True.

**Fig. 4:**
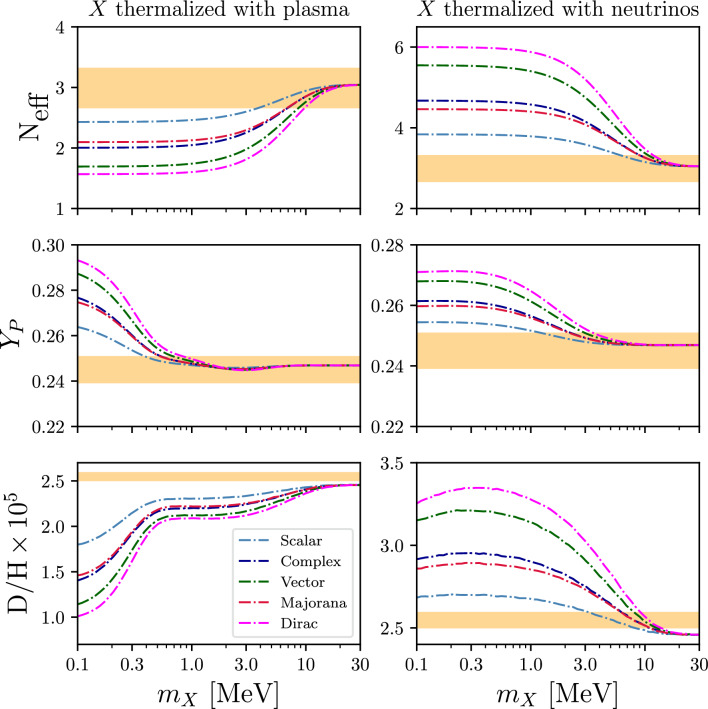
Investigation of the cosmological impact at the end of the BBN era from a new relativistic species *X* with degrees of freedom corresponding to a real/complex scalar (light/dark-blue lines), a real massive vector (magenta), or a Majorana/Dirac fermion (red/green); *X* is assumed to be in thermal equilibrium with either the electron-positron-photon plasma (left panels) or with the SM neutrino thermal bath (right panels). The orange bands represent the observational constraints at the 2$\sigma$ level from Refs. [24, 42]. Predictions with PRyMordialare obtained at nominal inputs and rates

In Fig. 4 we present the results for NP scenarios of this type, reproducing the qualitative features already well-discussed, e.g., in Ref. [31]. In particular, we observe three primary NP effects: (i) a change in the cosmological expansion rate, affecting the time-temperature relation; (ii) an impact on the evolution of the neutrino-to-photon temperature ratio, relevant for both neutrino and neutron decoupling; and (iii) additional entropy released in the plasma, altering the number of baryons per a given baryon-to-photon ratio. Note that in Fig. 4 we use the set of nuclear reactions from PRIMAT (nacreii_flag = False) and as a result a neutrinophilic species around $\sim 10~$MeV in mass appears to be favored by current observations of primordial *D*/*H* while remaining compatible with the other cosmological NP probes based on helium-4 and $N_\textrm{eff}$.

In contrast to the previous scripts, this code calls PRyMclass() with three functions (of temperature) as arguments: the contribution to the energy density, its derivative, and the pressure of the new species added to the bath. More generally, one can include a new interacting sector with its own temperature $T_\textrm{NP}$ and non-trivial collision term $\delta C_\textrm{NP}$ along the lines of the recent work in Ref. [34]. In PRyMordial one may study such “dark sectors” consistently by generalizing the set of equations in Eq. (3) to follow $T_\textrm{NP}$ together with $T_{\gamma , \nu }$, and solving for the entropy density involved in Eq (8) taking into account the effect of the NP. To do this, one switches on the Boolean flag NP_thermo_flag and codes all of the relevant contributions to the energy density, its derivative (which can optionally be evaluated numerically via Numdifftools), pressure and collision term for the NP sector, and passes them to PRyMresults.

One can also study NP resulting in changes to the weak rates for neutron freeze out and/or any of the implemented thermonuclear rates. To modify the weak rates, one sets the Boolean flag NP_nTOp_flag = True and change the parameter NP_delta_nTOp from its default of zero. Also, for the nuclear rates one switches on the flag NP_nuclear_flag and modifies the value of NP_delta_R with R being the reaction of interest.

As an example, we consider NP which results in a small change to the $n \leftrightarrow p$ conversion rates. We perform a Bayesian fit to $Y_{P}$ and *D*/*H* (as quoted by the PDG [42]) and allowing $\tau _\textrm{n}$, $\Omega _{B}h^2$, and the other key nuclear rates to vary within their uncertainties (in line with the SM MC analysis of the previous subsection):

This code can be simply generalized to modify any of the other nuclear reactions.

**Fig. 5:**
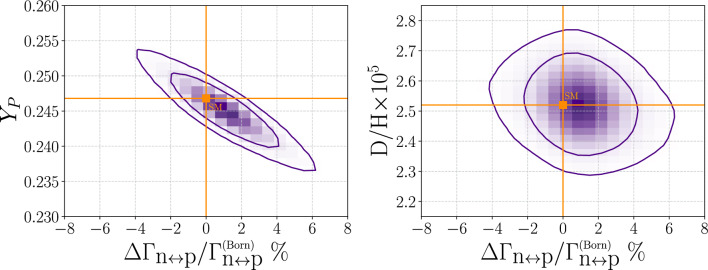
Constraint on a relative change of the weak $n \leftrightarrow p$ conversion rates from NP, based on a Bayesian fit performed with PRyMordial with the use of the emcee [56] package. Gaussian priors on the neutron lifetime and the cosmic baryon abundance are assumed (as for Fig. 3) and flags smallnet_flag *and* nacreii_flag are both switched on. Helium-4, deuterium measurements correspond to the recommended values from the PDG [42]

Figure 5 shows the resulting 2D joint (68% and 95%) probability regions for NP_delta_nTOp correlated with the measurements of primordial helium-4 and deuterium. To perform the statistical analysis, we adopt the emcee package [56]. For the sake of computational efficiency, we restrict the analysis to the network of 12 key reactions (with nacreii_flag = True), as is sufficient given the focus on helium-4 and deuterium. Figure 5 indicates that BBN is consistent with NP in the $n \leftrightarrow p$ conversion rates at the level of at most a few percent relative to the standard Born rates. The tight correlation with $Y_{P}$ illustrates the importance of neutron freeze out in determining the primordial helium-4 abundance.

## Outlook

In this work we have presented PRyMordial: A new tool to explore the physics of BBN in great detail, with an unprecedented eye toward applications for physics beyond the SM. The package also allows for fast, user-friendly precision analyses of the BBN era within the SM of Particle Physics, reaching the same level of accuracy as the state-of-the-art codes publicly available.

In Sect. 2 we provide in some detail a review of the BBN era, highlighting the physics in the code. The main novelties in PRyMordial are that it is:

- A package entirely written in Python, easy to install, run and modify, efficient in the evaluation of the key quantities for the study of BBN; moreover, an optional dependence on Julia allows the user to make the code run even faster;
- A computation of the thermal background based on the Boltzmann equations governing the evolution of the relativistic species present at that time. This allows for an accurate prediction of $N_\textrm{eff}$ from first principles and opens up new avenues for the study of BSM Physics;
- A fast and accurate evaluation of the weak rates including QED, nucleon-finite mass and thermal corrections for a prediction of the neutron-to-proton ratio that confronts the precision of current and next-generation measurements;
- A BBN code that easily allows exploration of uncertainties and changes in all of the input parameters and most importantly, includes by default different treatments for the nuclear rates in order to give to the user a better handle on the overall theoretical systematics.

In Sect. 3 we describe the structure of the code and provide examples of its usage within the Standard Model and for a few interesting scenarios of NP.

There are many directions that can be pursued in the future to make PRyMordial an even more compelling and flexible tool for the community. One important aspect we plan to expand upon is the characterization of the thermal background. At the moment, only a single common temperature for neutrinos is considered and no evolution equation for primordial chemical potentials is given by default. All of these can be easily implemented along the lines of Ref. [53].

Also relevant for precision studies would be an approach to efficiently include effects from phase-space spectral distortions of relativistic species. In this regard, we plan to further enrich the physics in PRyMordial with a dedicated framework for neutrino decoupling that includes effects from oscillations at non-zero lepton chemical potentials, see Ref. [110].

It would be a very interesting (though formidable) task to improve the current next-to-leading order computation of neutron freeze out in the Early Universe, filling in the gaps of some of the approximations undertaken in the literature (see Appendix B of  [111] as well as the improvements brought by the recent effective-field-theory study at zero temperature of Ref. [77]). We eventually plan to include higher-order QED corrections such as the ones available in Refs. [64] and [112], as well as the NLO QED corrections to $e^{+}e^{-} \leftrightarrow \nu \bar{\nu }$ matrix elements inspected in Ref. [113].

Finally, in the future we would like to enlarge the nuclear network beyond the 63 nuclear reactions currently implemented, which encode all of the processes involving nuclides up to boron-8 in atomic and mass number (needed for an accurate prediction of lithium-7 in the Standard Model).

With the public release of PRyMordial we hope to provide to the community an important new tool to address fundamental questions about the Early Universe, whose study remains central to further progress in our understanding of Nature. In the wise words of a giant of our time [1]:

*“[Human beings] are not content to comfort themselves with tales of gods and giants, or to confine their thoughts to the daily affairs of life; they also build telescopes and satellites and accelerators, and sit at their desks for endless hours working out the meaning of the data they gather. The effort to understand the universe is one of the very few things that lifts human life a little above the level of farce, and gives it some of the grace of tragedy.”*

**Note about referencing:** PRyMordial makes use of previous work in the literature. When using it, please be sure to appropriately reference the original literature as well as PRyMordial itself.

## Data Availability

This manuscript has associated data in a data repository. [Authors’ comment: All the results in this paper can be reproduced using the PRyMordial package, publicly available at https://github.com/vallima/PRyMordial.]

## Appendix A: How to install PRyMordial

PRyMordial is publicly released on GitHub. Once in the desired directory, from your terminal type:

The code requires a modern distribution of Python (Python 3 recommended) in order to properly run, and features only a couple of standard libraries as mandatory dependencies:

- NumPy (mandatory) – pip install numpy;
- SciPy (mandatory) – pip install scipy;
- Vegas (mandatory) – pip install vegas;
- Numba (recommended) – pip install numba;
- Numdifftools (recommended) – pip install numdifftools;
- PyJulia (optional) – pip install julia;
- diffeqpy (optional) – pip install diffeqpy.

Indeed, the code can easily avoid dependencies on Numba and Numdifftools. Giving up on Numba will slightly slow down a few routines in PRyM_thermo.py which involve SciPy integration. Also, the installation of Vegas library is required only in the case where thermal corrections to the weak rates governing neutron freeze out have to be recomputed. This is usually not the case, since those are already tiny effects in the SM and can be reasonably neglected in studies of NP during BBN.

The optional dependencies above require the Julia programming language to be installed. It can be downloaded at https://julialang.org. Once Julia is installed, it is recommended to create a soft link from the terminal typing something like:

Then, launch Julia and install DifferentialEquations.jl of the SciML kit (with Sundials wrapper): After a successful installation of the package, one needs to open a Python shell and type: At this point the user will be able to exploit the SciML routines developed in Julia to solve the nuclear-reaction network in PRyMordial, speeding up the execution of time by a factor of two or more, and with the possibility of cherry-picking from a large collection of differential-equation solvers built-in in the package, see the documentation here.

To use the SciML routines, the user must set the flag PRyM_init.flag_julia = True. In some systems, the very first call of PRyM_main. PRyMresults() might need to be in Python and therefore requires initially PRyM_init.flag _julia = False. Also, the first call in Julia will inevitably be slow, since it will compile PRyM_jl_sys.py. As a concise example of the dedicated script runPRyM_julia.py coming with the present release, here below is how things should work in the Julia mode:

**Table 1:** The key nuclear reactions adopted in PRyMordial, with corresponding references. The red (blue) column refers to the option nacreii_flag = True (False), see Sect. 2.3 for further details. Notice that the compilation of the blue column is present also in the code PRIMAT [47]

Nuclear reaction	References	References
n+p $\rightarrow$ D+$\gamma$	[114]	[114]
D+p $\rightarrow$ $^{3}$He+$\gamma$	[106]	[106]
D+D $\rightarrow$ $^{3}$He+n	[98]	[115]
D+D $\rightarrow$ $^{3}$H+p	[98]	[115]
$^{3}$H+p $\rightarrow$ $^{4}$He+$\gamma$	[92]	[92]
$^{3}$H+D $\rightarrow$ $^{4}$He+n	[96]	[115]
$^{3}$H+$^{4}$He $\rightarrow$ $^{7}$Li+$\gamma$	[96]	[115]
$^{3}$He+n $\rightarrow$ $^{3}$H+p	[96]	[102]
$^{3}$He+D $\rightarrow$ $^{4}$He+p	[96]	[115]
$^{3}$He+$^{4}$He $\rightarrow$ $^{7}$Be+$\gamma$	[98]	[115]
$^{7}$Be+n $\rightarrow$ $^{7}$Li+p	[96]	[103]
$^{7}$Li+p $\rightarrow$ $^{4}$He+$^{4}$He	[96]	[115]

## Appendix B: Nuclear processes in PRyMordial

**Table 2:** Nuclear processes beyond the key ones implemented in the package PRyMordial, with related references. Those processes are particularly needed for a precise prediction of the primordial abundance of lithium-7. Notice that the compilation above is part of the larger one present in the code PRIMAT [47]

Nuclear reaction	References
$^{7}$Li+p $\rightarrow$ $^{4}$He+$^{4}$He+$\gamma$	[115]
$^{7}$Be+n $\rightarrow$ $^{4}$He+$^{4}$He	[116]
$^{7}$Be+D $\rightarrow$ $^{4}$He+$^{4}$He+p	[117]
D+$^{4}$He $\rightarrow$ $^{6}$Li+$\gamma$	[118]
$^{6}$Li+p $\rightarrow$ $^{7}$Be+$\gamma$	[115]
$^{6}$Li+p $\rightarrow$ $^{3}$He+$^{4}$He	[115]
$^{8}$B+n $\rightarrow$ $^{4}$He+$^{4}$He+p	[119]
$^{6}$Li+$^{3}$He $\rightarrow$ $^{4}$He+$^{4}$He+p	[119]
$^{6}$Li+$^{3}$H $\rightarrow$ $^{4}$He+$^{4}$He+n	[119]
$^{6}$Li+$^{3}$H $\rightarrow$ $^{8}$Li+p	[119]
$^{7}$Li+$^{3}$He $\rightarrow$ $^{6}$Li+$^{4}$He	[119]
$^{8}$Li+$^{3}$He $\rightarrow$ $^{7}$Li+$^{4}$He	[119]
$^{7}$Be+$^{3}$H $\rightarrow$ $^{6}$Li+$^{4}$He	[119]
$^{8}$B+$^{3}$H $\rightarrow$ $^{7}$Be+$^{4}$He	[119]
$^{8}$B+n $\rightarrow$ $^{6}$Li+$^{3}$He	[119]
$^{8}$B+n $\rightarrow$ $^{7}$Be+D	[119]
$^{6}$Li+$^{3}$H $\rightarrow$ $^{7}$Li+D	[119]
$^{6}$Li+$^{3}$He $\rightarrow$ $^{7}$Be+D	[119]
$^{7}$Li+$^{3}$He $\rightarrow$ $^{4}$He+$^{4}$He+D	[119]
$^{8}$Li+$^{3}$He $\rightarrow$ $^{4}$He+$^{4}$He+$^{3}$H	[119]
$^{7}$Be+$^{3}$H $\rightarrow$ $^{4}$He+$^{4}$He+D	[119]
$^{7}$Be+$^{3}$H $\rightarrow$ $^{7}$Li+$^{3}$He	[119]
$^{8}$B+D $\rightarrow$ $^{7}$Be+$^{3}$He	[119]
$^{8}$B+$^{3}$H $\rightarrow$ $^{4}$He+$^{4}$He+$^{3}$He	[119]
$^{7}$Be+$^{3}$He $\rightarrow$ p+p+$^{4}$He+$^{4}$He	[119]
D+D $\rightarrow$ $^{4}$He+$\gamma$	[115]
$^{3}$He+$^{3}$He $\rightarrow$ $^{4}$He+p+p	[115]
$^{7}$Be+p $\rightarrow$ $^{8}$B+$\gamma$	[115]
$^{7}$Li+D $\rightarrow$ $^{4}$He+$^{4}$He+n	[120]
D+n $\rightarrow$ $^{3}$H+$\gamma$	[121]
$^{3}$H+$^{3}$H $\rightarrow$ $^{4}$He+n+n	[121]
$^{3}$He+n $\rightarrow$ $^{4}$He+$\gamma$	[90]
$^{3}$He+$^{3}$H $\rightarrow$ $^{4}$He+D	[117]
$^{3}$He+$^{3}$H $\rightarrow$ $^{4}$He+n+p	[117]
$^{7}$Li+$^{3}$H $\rightarrow$ $^{4}$He+$^{4}$He+n+n	[117, 122]
$^{7}$Li+$^{3}$He $\rightarrow$ $^{4}$He+$^{4}$He+n+p	[117, 122]
$^{8}$Li+D $\rightarrow$ $^{7}$Li+$^{3}$H	[123]
$^{7}$Be+$^{3}$H $\rightarrow$ $^{4}$He+$^{4}$He+n+p	[117, 122]
$^{7}$Be+$^{3}$He $\rightarrow$ $^{4}$He+$^{4}$He+p+p	[117, 122]
$^{6}$Li+n $\rightarrow$ $^{3}$H+$^{4}$He	[117]
$^{3}$He+$^{3}$H $\rightarrow$ $^{6}$Li+$\gamma$	[124]
$^{4}$He+n+p $\rightarrow$ $^{6}$Li+$\gamma$	[117]
$^{6}$Li+n $\rightarrow$ $^{7}$Li+$\gamma$	[122]
$^{6}$Li+D $\rightarrow$ $^{7}$Li+p	[122]
$^{6}$Li+D $\rightarrow$ $^{7}$Be+n	[122]
$^{7}$Li+n $\rightarrow$ $^{8}$Li+$\gamma$	[122, 125]
$^{7}$Li+D $\rightarrow$ $^{8}$Li+p	[122]
$^{8}$Li+p $\rightarrow$ $^{4}$He+$^{4}$He+n	[126]
$^{4}$He+n+n $\rightarrow$ $^{6}$He+$\gamma$	[127]
p+p+n $\rightarrow$ D+p	[117]
$^{7}$Li+$^{3}$H $\rightarrow$ $^{4}$He+$^{4}$He+n+n	[117, 122]

In this appendix we collect the 12 key reactions necessary to accurately predict helium-4 and deuterium, see Table 1, as well as the 51 additional reactions comprising the full set recommended for a more robust prediction of lithium-7, Table 2. For the general aspects of the evaluation of the nuclear rates in the Early Universe as well as the theoretical and statistical details behind the compilation of the nuclear rates present in PRyMordial, we refer the interested reader to Refs. [92, 107, 108].

## Footnotes

1 While $T_{e} = T_{\gamma }$ follows from $e^{\pm }$ being tightly coupled to photons via fast QED processes, the approximation underlying $T_{\nu }$, namely $T_{\nu _{e}} \simeq T_{\nu _{\mu }} \simeq T_{\nu _{\tau }}$, can be motivated by the active flavor mixing of $\nu$ oscillations at $T_{\gamma }$ of few MeV [62].

2 In the current version of PRyMordial we adopt the computation of $\delta C_{\nu }$ as well as the next-to-leading (NLO) QED corrections to the electromagnetic pressure of the plasma directly from the numerical results tabulated in NUDEC_BSM [53].

3 Equation (3) can be easily generalized to include new sectors. This contrasts with typical existing BBN codes which compute the thermodynamic background by interpolating the tabulated result of the (numerically intensive) integro-differential Boltzmann equation, solved for the neutrino phase-space density in the SM.

4 Notice that in absence of interactions for the species *i*, entropy conservation can be guaranteed either by a negligible chemical potential, $\mu _{i} \ll T_{i}$ or by number density conservation per comoving volume, $d(n_{i} a^3)/dt = 0$.

5 $\mu _{e}/T_{\gamma } \ll 1$ is justified in the SM by $\eta _{B}\sim \mathcal {O}(10^{-10})$ and the condition of electric charge neutrality in the Early Universe.

6 Any treatment must confront both the *neutron lifetime puzzle* i.e. the tension between “bottle” [78] and “beam” [79] measurements of $\tau _\textrm{n}$, see, e.g., [80]; and the *Cabibbo angle anomaly* [81], i.e. the extraction of $V_\textrm{ud}$ from super-allowed $\beta$ decays and $V_\textrm{us}$ from semi-leptonic decays versus unitarity in the Cabibbo–Kobayashi–Maskawa matrix [72].

7 In the current version of PRyMordial we include up to boron-8 in the nuclear chains, which is sufficient for an accurate prediction of lithium-7, likely the heaviest nuclide of interest when confronting BBN with observations [94]. For this purpose, the largest implemented set of thermonuclear rates comprises 63 reactions, see Appendix B.

8 Notice that this definition differs at the sub-percent level from the helium mass fraction adopted in the context of the CMB [24]: $Y_{P}:^\textrm{CMB} \equiv (m_\mathrm{^4 He}/4) \, Y_{P}/[(m_\mathrm{^4 He}/4) Y_{P} + m_\textrm{H} \, (1-Y_{P})]$, with $m_\mathrm{H, ^4 He}$ the atomic mass of hydrogen and helium.

9 This fact has been more quantitatively acknowledged in Ref. [35] which used a beta version of PRyMordial The impact of LUNA has been explored for the first time in Refs. [48–50].

10 The corresponding reverse reactions are obtained via Eq. (20) from the interpolated forward rates.

11 For the electroweak sector we adopt $\{\alpha _\textrm{em};$, $G_{\textrm{F}}$, $M_\textrm{Z}\}$ as inputs and derive the rest via tree-level relations.

12 The results in Fig. 3 slightly differ from Ref. [35] due to an update on the Gaussian prior for the neutron lifetime and the different choice for the cosmological baryon abundance adopted in that study.
